# Linking Dementia Pathology and Alteration in Brain Activation to Complex Daily Functional Decline During the Preclinical Dementia Stages: Protocol for a Prospective Observational Cohort Study

**DOI:** 10.2196/56726

**Published:** 2024-06-06

**Authors:** Pierfilippo De Sanctis, Jeannette R Mahoney, Johanna Wagner, Helena M Blumen, Wenzhu Mowrey, Emmeline Ayers, Claudia Schneider, Natasha Orellana, Sophie Molholm, Joe Verghese

**Affiliations:** 1 Department of Neurology, Division of Cognitive and Motor Aging Albert Einstein College of Medicine Bronx, NY United States; 2 Department of Pediatrics, Cognitive Neurophysiology Laboratory Albert Einstein College of Medicine Bronx, NY United States; 3 Swartz Center for Computational Neuroscience Institute for Neural Computation University of California San Diego, La Jolla, CA United States; 4 Department of Medicine (Geriatrics) Albert Einstein College of Medicine Bronx, NY United States; 5 Department of Epidemiology and Population Health Albert Einstein College of Medicine Bronx, NY United States

**Keywords:** EEG, electroencephalographic, mobility, preclinical dementia stages

## Abstract

**Background:**

Progressive difficulty in performing everyday functional activities is a key diagnostic feature of dementia syndromes. However, not much is known about the neural signature of functional decline, particularly during the very early stages of dementia. Early intervention before overt impairment is observed offers the best hope of reducing the burdens of Alzheimer disease (AD) and other dementias. However, to justify early intervention, those at risk need to be detected earlier and more accurately. The decline in complex daily function (CdF) such as managing medications has been reported to precede impairment in basic activities of daily living (eg, eating and dressing).

**Objective:**

Our goal is to establish the neural signature of decline in CdF during the preclinical dementia period.

**Methods:**

Gait is central to many CdF and community-based activities. Hence, to elucidate the neural signature of CdF, we validated a novel electroencephalographic approach to measuring gait-related brain activation while participants perform complex gait-based functional tasks. We hypothesize that dementia-related pathology during the preclinical period activates a unique gait-related electroencephalographic (grEEG) pattern that predicts a subsequent decline in CdF.

**Results:**

We provide preliminary findings showing that older adults reporting CdF limitations can be characterized by a unique gait-related neural signature: weaker sensorimotor and stronger motor control activation. This subsample also had smaller brain volume and white matter hyperintensities in regions affected early by dementia and engaged in less physical exercise. We propose a prospective observational cohort study in cognitively unimpaired older adults with and without subclinical AD (plasma amyloid-β) and vascular (white matter hyperintensities) pathologies. We aim to (1) establish the unique grEEG activation as the neural signature and predictor of decline in CdF during the preclinical dementia period; (2) determine associations between dementia-related pathologies and incidence of the neural signature of CdF; and (3) establish associations between a dementia risk factor, physical inactivity, and the neural signature of CdF.

**Conclusions:**

By establishing the clinical relevance and biological basis of the neural signature of CdF decline, we aim to improve prediction during the preclinical stages of ADs and other dementias. Our approach has important research and translational implications because grEEG protocols are relatively inexpensive and portable, and predicting CdF decline may have real-world benefits.

**International Registered Report Identifier (IRRID):**

DERR1-10.2196/56726

## Introduction

### Background

Progressive difficulty in performing everyday functional activities is a key diagnostic feature of dementia. Yet not much is known about the neural underpinnings of functional decline, particularly during the very early stages of dementia. Early intervention before impairment in everyday functions is observed offers the best hope of reducing the burdens of Alzheimer disease (AD) and other dementias. However, to justify early intervention, those at risk need to be detected earlier and more accurately.

The decline in complex daily function (CdF) such as managing medications and finances has been reported to precede impairment in basic activities of daily living (eg, eating and dressing). Our goal is to establish the neural signature of decline in CdF during the preclinical dementia period. We recently reported that limitations in CdF were more prevalent or declined faster in those who converted to mild cognitive impairment (MCI) [1]. We compared CdF profiles at baseline in 59 community-dwelling older individuals with normal cognitive performance who went on to develop incident MCI (“pre-MCI”) with 284 older individuals who remained cognitively normal over follow-up. While limitations in CdF were reported to be subtle, the mean number of limitations at baseline was 3.1 (SD 3.0) in the pre-MCI cases and 2.0 (SD 2.4) in normal controls (*P*=.003). This is in line with studies using amyloid-β (Aβ), phosphorylated τ, neurofilament (also referred to as AT(N) classification system) [2] to stratify dementia risk in cognitively normal older adults, showing the presence of AT(N) biomarkers to be associated with more limitations in CdF [3,4].

While combining behavioral (eg, cognition and CdF assessment) with biological (eg, AT(N)) markers has further improved early risk assessment, it is insufficient to justify intervention during the preclinical dementia stages. For example, up to one-third of older individuals with significant accumulation of AD-related and vascular pathology will not progress to MCI and dementia stages [5,6]. In this proposal, we seek to further improve risk assessment by establishing the neural signature of CdF. We posit that abnormal patterns of neurophysiological activation precedes and can serve as a robust predictor of change in CdF, which in turn increases the risk of conversion to dementia [7]. To test this hypothesis, we designed complex gait tasks. Gait has physical and cognitive contributions. We showed that gait performance (eg, gait speed) during normal and dual-task conditions is associated with scores on the Activities of Daily Living–Prevention Instrument (ADL-PI) [8], which includes questions about abilities to manage money, drive, do the laundry, and use appliances [9,10]. We applied a novel electroencephalographic-based mobile brain-body imaging approach to measure gait-related brain activation while participants perform complex gait-based functional tasks [11-19]. Raising the complexity of the gait task may allow early distinction of individuals with and without the risk of developing limitations in CdF by increasing the cognitive demands of the task. We designed simple (walking-only) and complex (dual-task walking) gait-based tasks to measure differential activation (complex minus simple gait). This proposal builds on and extends 2 decades of work by our team showing that changes in gait (eg, slowing of walking speed) during simple and complex walking conditions are robust predictors of decline in CdF, cognitive decline, and progression to dementia [9,10,20-30].

### Hypothesis

**Figure 1:**
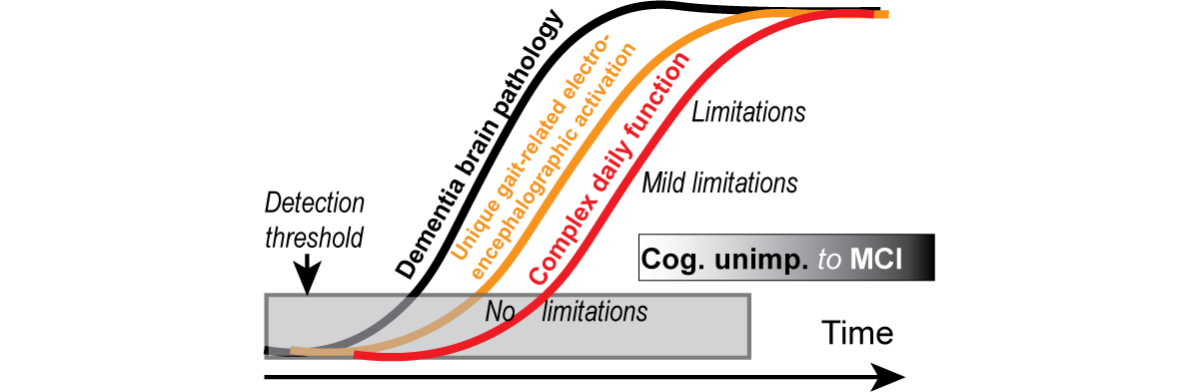
Sequence of events leading to decline in CdF. CdF: complex daily function; Cog.: cognitive; MCI: mild cognitive impairment; Unimp.: unimpaired.

Our overarching hypothesis illustrated in Figure 1 is that the presence of dementia-related pathology during the preclinical period of dementia is associated with a unique gait-related electroencephalographic (grEEG) pattern that predicts a subsequent decline in CdF. The first aim of this study is to establish the neural signature of CdF. Our preliminary findings show that individuals reporting CdF limitations can be characterized by weaker pre as well as postcentral gyrus and stronger fronto-medial activation during complex gait (eg, dual-task walking). This unique grEEG activation pattern (neural signature) will predict CdF decline longitudinally. Our second aim is to determine the association of dementia-related pathology with the incidence of the neural signature of CdF in individuals without the neural signature at baseline. Specifically, we hypothesize that plasma-based assays of Aβ, T, and white matter hyperintensities (WMH) at baseline will independently and in combination predict the incidence of the neural signature of CdF. We also need to understand if and how behaviors are known to increase dementia risk (eg, physical inactivity) and may modify the neural signature of CdF as a prelude to developing interventions. Our third aim is to determine whether physical inactivity is associated with the prevalence and incidence of the neural signature of CdF in our sample.

This project is part of a larger effort at the Division of Cognitive and Motor Aging at Albert Einstein College of Medicine (AECOM) to go beyond memory-centered depictions of AD and consider cognitive processes related to motor and sensory abilities as important additional characterization of AD and related dementias. We are partnered with an ongoing investigation (R01AG075679-01) that seeks to establish visual-somatosensory integration (VSI study) as a novel marker of preclinical AD. With Aβ accumulation occurring early in sensory association areas [31], the VSI study (principal investigator [PI] JRM) seeks to define multisensory integration as a preclinical marker of AD and related dementias (see [32] for more details). The VSI study serves as a parent study for cross-enrollment and includes older adults across the cognitive spectrum from cognitively normal to MCI. Participants in the VSI study with confirmed preclinical AD diagnosis (defined as Aβ positive) will undergo positron emission tomography testing. Collectively, these studies will broaden the scope of our investigations in synergistic ways to deepen our understanding of the very early preclinical dementia period.

## Methods

### Study Design and Recruitment

**Figure 2:**
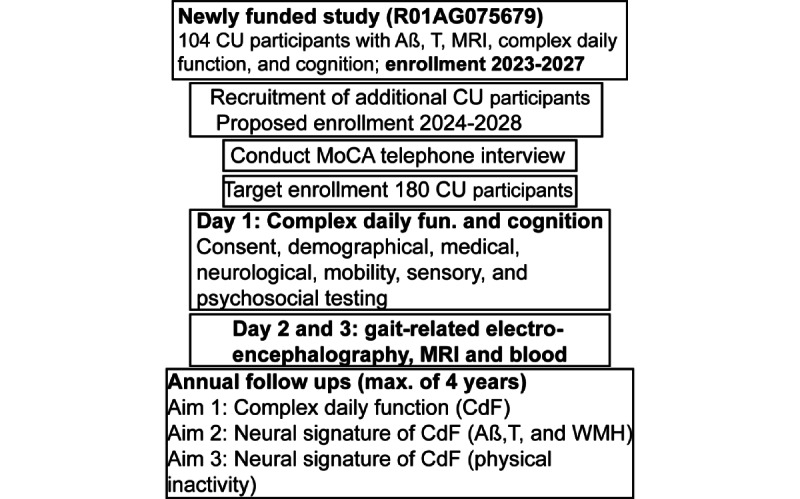
Recruitment and procedures conducted under this proposal. Aβ: amyloid beta; CdF: complex daily function; CU: cognitively unimpaired; fun.: function; max.: maximum; MoCA: Montreal Cognitive Assessment; MRI: magnetic resonance imaging; T: phosphorylated τ; WMH: white matter hyperintensities.

We propose a prospective observational cohort study of 180 older adults who are cognitively unimpaired. Figure 2 shows the recruitment and timeline of procedures. We will cross-enroll participants from an active study (R01AG075679, PI JRM). We will use telephone-based screening procedures to enroll and follow a community-based cohort. Potential participants aged 65 year and older from the greater New York City area are first contacted by mail and then by telephone to explain the purpose and nature of this study. The telephone interview includes verbal consent, a brief medical history questionnaire, and 2 brief cognitive screens [33,34]. We will ensure that enrollment reflects the race and ethnic diversity of our catchment area. Following the interview, potential participants are invited for further evaluation at our research center. If eligible, written informed consents are obtained at study visits as per institutional review board (IRB) guidelines. This study is approved by the AECOM IRB.

### Study Criteria

#### Inclusion Criteria

Adults are included who are aged 65 years and older, can speak and write in English, reside in the New York metropolitan area, plan to be in the area for the next 3 or more years, and are ambulatory.

#### Exclusion Criteria

Exclusion criteria were previous MCI or dementia diagnosis, reported severe sensory impairments, chronic medication use (eg, neuroleptics) that influence cognitive functioning, terminal illness with life expectancy <12 months, existing diagnosis of neurodegenerative diseases (eg, Parkinson disease or amyotrophic lateral sclerosis), presence of clinical disorders that overtly alter attention like delirium, or major psychiatric disorder such as schizophrenia. Additional electroencephalographic and magnetic resonance imaging (MRI)–specific exclusion criteria are seizure medication, stroke, traumatic brain injury, claustrophobia, and MRI contraindications such as pacemakers or any permanent magnetic metal implants.

### Assessments

Table 1 lists instruments used to assess function and other domains. The ADL-PI will serve as our primary outcome measure. It is a 15-item questionnaire that was developed to target the earliest changes in complex daily activities [8]. A performance-based instrument, the Everyday Problems Test [35], will serve as our secondary CdF outcome measure. It is a 42-item paper-pencil test that examines abilities to understand and execute complex daily activities (eg, write a check).

**Table 1:** Measurements conducted under this proposal.

Domain and measures				Time
**Independent variables**				
	Aim 1: neural signature of CdF^a^ (mobile electroencephalographic)		2.5 h including rest	
	Aim 2: MRI^b^ white matter hyperintensity and plasma-based Aβ42^c^/Aβ40/τ		1 h	
	Aim 3: Physical Activity Scale for the Elderly [36] and accelerometry		5 min and 7-d period	
**Outcomes (yearly assessed)**				
	Aim 1: Activities of Daily Living–Prevention Instrument [8]—primary		5 min	
	Aim 1: Everyday Problem Test—secondary		45 min	
	Aims 2 and 3: Incident neural signature of CdF		2.5 h including rest	
**Covariates**				
	Cognition (moderator)	Montreal Cognitive Assessment [37] (moderator aim 1) Wechsler Adults Intelligence Scale IV [38]	55 min	
	Physical function	Quantitative Gait Assessment (ProtoKinetics Movement Analysis Software) [39-41], hand grip strength [42]	15 min	
	Psychosocial	Geriatric Depression Scale [43]; Beck Anxiety Inventory [44]	15 min	
	Dementia risk factors	*ApoE*, obesity, diet [45], social isolation [46], cognitive engagement [47], depression	30 min	
	Medical	Medical history, comorbidities, or medication [30,48]	20 min	
	Sensory	Visual sensory screen [49], Shoebox auditory testing, Vibratron [50], Michigan Neuropathy Screening Instrument [51,52]	15 min	

^a^CdF: complex daily function.

^b^MRI: magnetic resonance imaging.

^c^Aβ: amyloid-β.

Neuroimaging and plasma-based blood testing will be conducted during baseline visits for each participant. C2N Diagnostic will quantify plasma τ217 (nonphosphorylated and phosphorylated) and plasma amyloid (Aβ42, Aβ40), and provide plasma *ApoE* prototyping using liquid chromatography-tandem mass spectrometry platforms (PrecivityAD) [53-55]. Blood samples will be collected and stored in the biorepository at AECOM. Physical activity will be assessed at baseline and during annual in-person visits using the Physical Activity Scale for the Elderly [36] as well as objectively using accelerometry [56] over 7 days (AX3; Axivity Ltd). The Physical Activity Scale for the Elderly [57,58] and AX3 [59] are reliable and valid measures of physical activity.

### Cognitive Diagnoses

Established clinical consensus case conference procedures [60], where participants’ demographic, neuropsychological, neurological, psychosocial, and functional test results are reviewed by a multidisciplinary clinical team consisting of neurologists and neuropsychologists, will be used to determine normal cognitive status. Cognitively unimpaired individuals testing positive for Aβ are classified as preclinical AD [2]. Cognitive status—normal, MCI, and dementia—will be assigned at baseline and during yearly follow-up visits. Individuals with MCI and dementia at baseline are excluded from this project.

### Outcomes

For aim 1, the primary outcome is complex daily functional limitations measured with the ADL–PI, as reported in our previous publication [8]. For aims 2 and 3, the outcome is the incidence of the neural signature of CdF (see section below for definition). All outcome measures are assessed annually over a maximal period of 4 years.

### Ethical Considerations

Protocols for the proposed research project are approved by the AECOM’s IRB (2023-14773). Before enrollment, written consent is obtained from all interested persons who meet the inclusion criteria at the time of the visit. Confidentiality will be preserved by the use of unique ID codes for identification. ID and name associations will be password-protected in an encrypted master file to which only the PI and study coordinator have access. Participant data, including computer data disks, will be kept in a locked room. Participants will receive monetary compensation (US $20 per hour), free lunch, and transportation for participating in this study.

### grEEG Protocol

**Figure 3:**
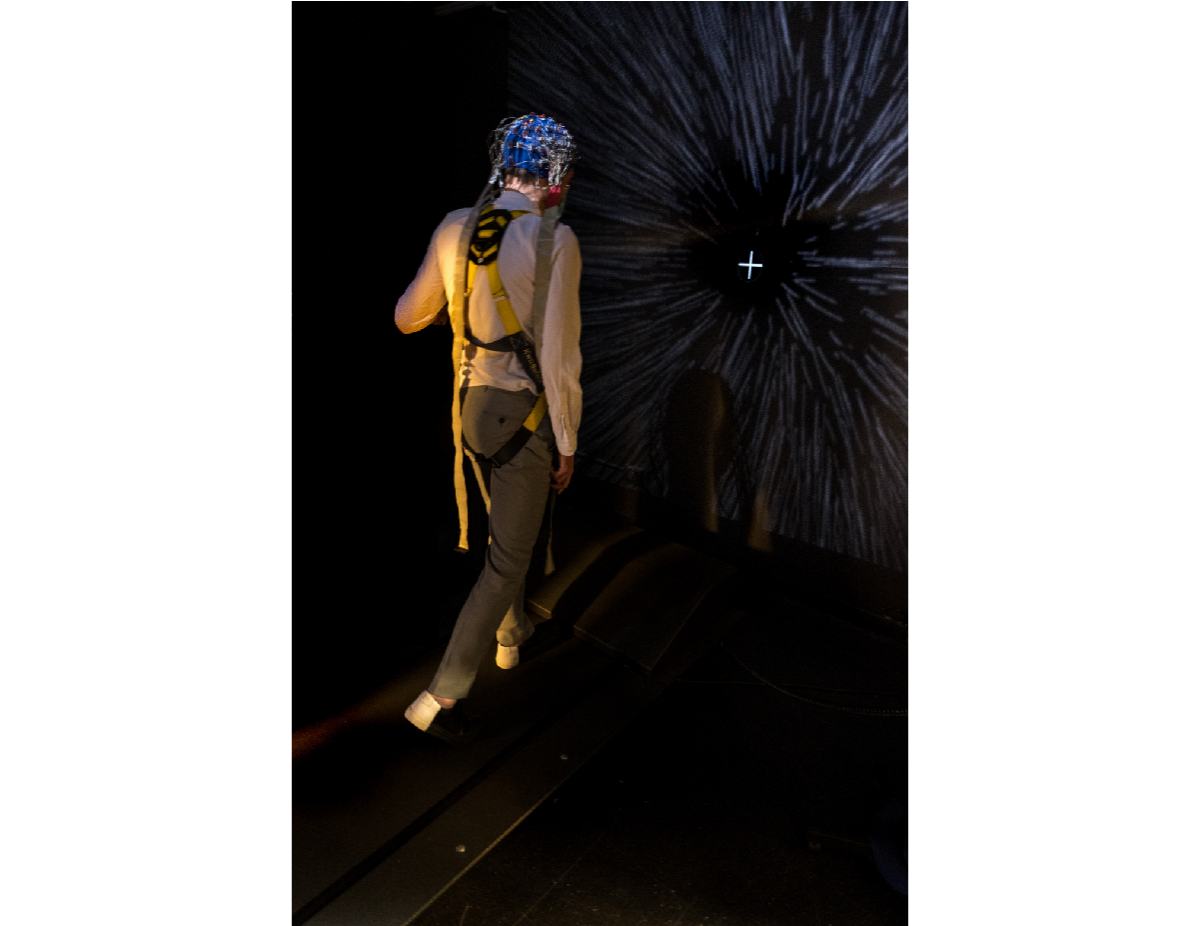
Participant during the visually perturbed walking task.

We designed simple (walking-only) and complex (dual-task walking) gait tasks to measure differential activation (complex minus simple gait). We provide preliminary findings showing that individuals reporting CdF limitations can be characterized by a unique grEEG pattern: weaker sensorimotor activation, measured by pre or postcentral gyrus 13-28 Hz desynchronization, and stronger motor control activation, measured by fronto-medial 3-7 Hz synchronization. Figure 3 shows an individual performing the visual-perturbed walking task. The participant walking on a treadmill wearing an electroencephalographic cap is immersed into a large-scale visual flow field designed to destabilize posture [61]. We superimpose sideway shifts onto a display of dots radiating outward from a central point of extension to create visual perturbations. We validated the task in prior studies [12,15]. Participants wore harnesses for safety.

### Neural Signatures—Starting With Cognition and Switching to CdF

In our first attempt to identify the neural signature of CdF, we stratified individuals by their cognitive performance [19]. Cognitively normal individuals (Montreal Cognitive Assessment [MoCA] ≥ 22 [62,63]) were dichotomized into low (score of 22-26; n=10) and high (score 27+; n=16) performing groups. We discovered a unique grEEG activation pattern time-locked to the gait cycle in low-cognitive performers: weaker central gyrus β (13-28 Hz) desynchronization paired with stronger fronto-medial θ (3-7 Hz) synchronization during visually perturbed compared to unperturbed walking. We also found correlations between θ synchronization and worse MoCA scores and between β desynchronization and better MoCA scores. Amplitude suppression (ie, desynchronization) of brain oscillatory activity within 8 to 28 Hz over pre as well as postcentral gyrus during movement is considered a neurophysiological marker of sensorimotor activation related to preparation and execution of movement [64-70]. We suggest that posterior parietal and sensorimotor activation during gait adjustment is related to prioritizing inputs from visual, somatosensory, and vestibular systems based on reliability to build accurate proprioception subserving motor output [19]. On the other hand, increased θ activity (ie, synchronization) over the fronto-medial cortex is thought of as a mechanism by which the need for cognitive or motor control is realized and signaled across brain regions [71,72]. In the context of movement, we and others observed fronto-medial θ in moments of postural instability [12,15,73-78].

### Neural Signature of CdF

To test whether this unique grEEG activation pattern also associates with the limitation in CdF, we dichotomized our sample in the following way: (1) compute the difference in activation between unperturbed and perturbed walking; (2) use inverse source localization techniques to estimate intracranial sources of scalp-recorded activity; (3) separate individuals with source localized to fronto-medial regions into groups of weak and strong θ synchronizers using the median; (4) separate individuals without sources in fronto-medial and with sources in sensorimotor region into groups of weak and strong β desynchronizers using the median (we chose right sensorimotor β because effects where strongest for that region and frequency: *P*=.04) [19].

**Figure 4:**
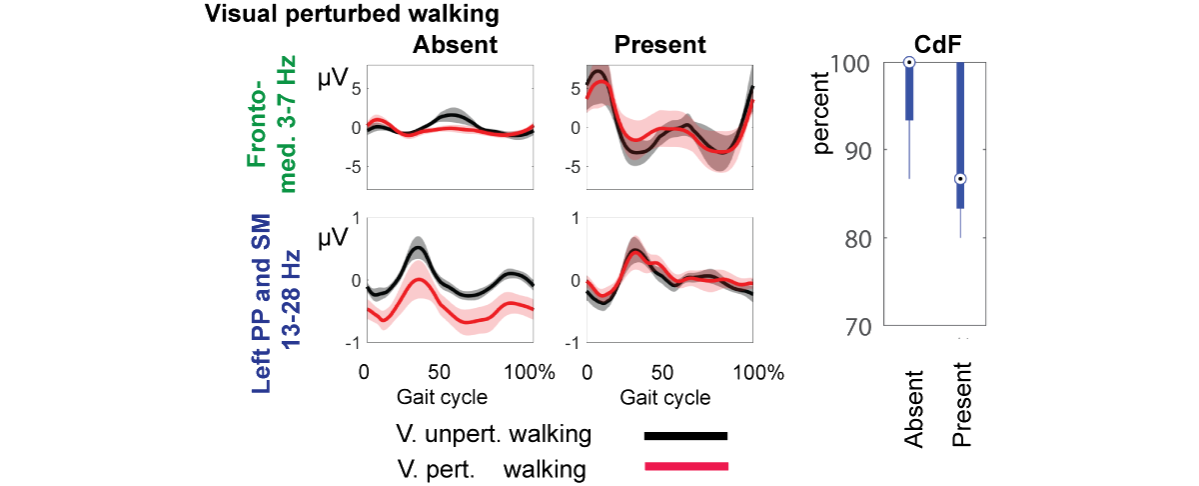
The neural signature of limitations in CdF and unique gait-related electroencephalographic activation. CdF: complex daily function; med.: medial; pert.: perturbed; PP: posterior parietal; SM: sensorimotor; unpert.: unperturbed; V.: visually.

Figure 4 shows preliminary findings in support of our hypothesis: Individuals characterized by a unique grEEG pattern report limitations in CdF to a higher degree.

## Results

### Aim 1—Establish the Neural Signature of CdF

#### Overview

**Figure 5:**
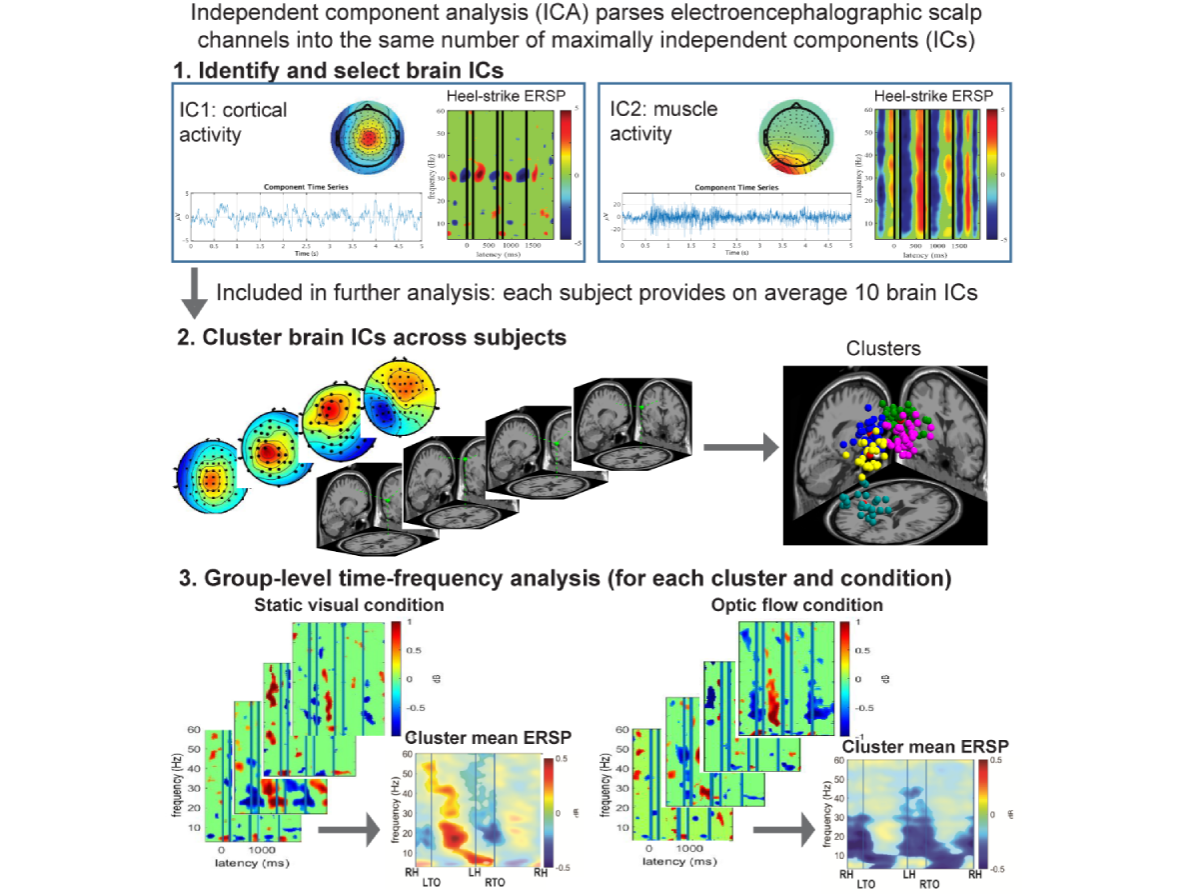
Analysis pipeline for separating brain activity from non–brain related artifacts during MoBI recording. ERSP: event-related spectral perturbation; IC: independent components; ICA: independent component analysis; LH: left heel strike; LTO: left toe off; MoBI: mobile brain body imaging; RH: right heel strike; RTO: right toe off.

Figure 4 shows individuals with and without the unique grEEG pattern and associations with mild complex daily functional limitations measured with ADL-PI [8,79]. A score of 100% in Figure 5 indicates all activities are performed without difficulties. In both walking tasks, individuals with the unique grEEG pattern expressed nominally more difficulties performing complex daily activities.

#### Walking Tasks

Participants will familiarize themselves with walking on the treadmill and are asked to determine their preferred walking speed before starting the experiment. For the VPW, participants will perform 4 visual perturbed and 4 unperturbed walking blocks randomized within participants. During perturbed stimulation, a large-scale visual field of dots is projected centrally onto a black wall in front of the participant. The stimulation consisted of 200 randomly placed white dots emanating outward from a central area of expansion. Superimposed to the outward motion is a sinusoidal perturbation in the mediolateral direction. A static image of dots placed randomly across the visual field serves as a control condition (ie, unperturbed walking). For the DTW, participants will perform the Go/No-Go task standing (4 blocks) and walking (4 blocks). Blocks are randomized within participants. The Go/No-Go stimuli consist of images from the International Affective Picture System [80]. Participants were instructed to click a wireless computer mouse button with the right hand each time a new image appeared (Go trials) but to withhold their response if the same image was shown twice in a row (No-Go trials). Each block consists of 180 trials and the probability of Go and No-Go trials will be 0.85 and 0.15, respectively. They will also walk wearing a safety harness.

#### Kinematics Recordings

The Optitrack infrared motion capture system with 9 cameras is used to collect kinematic data in the X/Y/Z direction at a sample frequency of 100 Hz (Arena version 1.5 acquisition software, Natural Point).

#### Electrophysiological Recordings

Continuous electroencephalography is recorded with a 64-channel BioSemi ActiveTwo system (digitized at 512 Hz; 0.05 to 100 Hz passband, 24 dB/octave). Data are high-pass filtered at 2 Hz and an automatic channel rejection procedure [81] is applied to exclude noise channels followed by visual inspection. Time-synchronized acquisition of electroencephalographic and motion tracking is conducted with Lab Streaming Layer software (Swartz Center for Computational Neuroscience, University of California San Diego).

#### Prevalence of Unique grEEG Activation

To estimate the prevalence of grEEG abnormalities in aging, we reanalyzed previously published data [16]. Using event-related potentials measured in control participants (ie, young adults), we defined a threshold (1.5 SD from the mean 2.2 µV) to calculate the percentage of unique grEEG (event-related potential, ERP_young_>1.5 SD, mean 2.2 µV) in older participants. The prevalence of grEEG abnormality in older adults was 25% (4 out of 16).

#### Data Analysis

##### Gait Kinematics

Spatiotemporal features of gait kinematics are determined by computing the velocity profile for each foot (for a detailed description, see De Sanctis et al [19]).

##### Integrated Gait-Electroencephalographic Analysis

Figure 5 shows the basic steps to identify or correct artifacts and model spatially resolved brain dynamics tied to the gait cycle that we apply in our proposal. We have validated and applied this analysis pipeline in prior work [12,15,61]. Independent component (IC) analysis will be used to parse electroencephalographic signals into ICs. The resulting ICs are source-localized using inverse source modeling [82,83]. Only dipoles located within the brain, with a fit accounting for at least 80% of the variance for a given IC scalp projection, are retained [84,85]. Cortical IC clustering will be used to cluster brain ICs across subjects using feature vectors coding for IC differences in power spectral density, dipole location, and scalp projection [86,87]. Event-related spectral perturbations (ERSPs) are computed from single trial spectrograms for each IC, time-locked to the right heel strike [88]. ERSPs are computed by determining the power spectra over a sliding latency window and normalizing the spectrogram by their respective mean spectra (averaged across the latency window) [88]. To average across all strides taken and compute gait cycle ERSPs, we time-warped single trial spectrograms applying a linear interpolation function to align left toe off, left heel strike, right toe off, and right heel strike across epochs following the methods introduced by Gwin and colleagues [87].

#### Statistical Plan Aim 1

##### Primary Analyses

grEEG measures will be compared between individuals with and without limitations in CdF using the 2-sample *t* tests to assess which grEEG measure is associated with limitations in CdF. Pearson correlation will be computed between grEEG measures and CdF scores.

##### Longitudinal Analyses

The time-to-event analyses including Kaplan-Meier survival curves, log-rank tests, and Cox proportional hazards regression models will be used to assess whether unique grEEG patterns predict a decline in CdF longitudinally.

##### Power Analysis

With the sample size of 180 older adults who are cognitively unimpaired, we estimate that there will be 54 individuals classified with CdF limitations based on our preliminary studies. With a significance level (α) of .05, using a 2-sided 2-sample *t* test, this study achieves 80% power to detect a minimal difference of 0.5 SD in the grEEG measure between CdF limitation groups. The minimum detectable hazards ratio for CdF decline between older adults with and without unique grEEG pattern ranges from 1.52 to 1.69 (assuming event rate between 0.30 and 0.48) with a power of 0.8 and a 2-sided type I error rate of 5% [89].

### Aim 2—Determine Association of Dementia-Related Pathology With Incidence of Neural Signature of CdF

#### Overview

**Figure 6:**
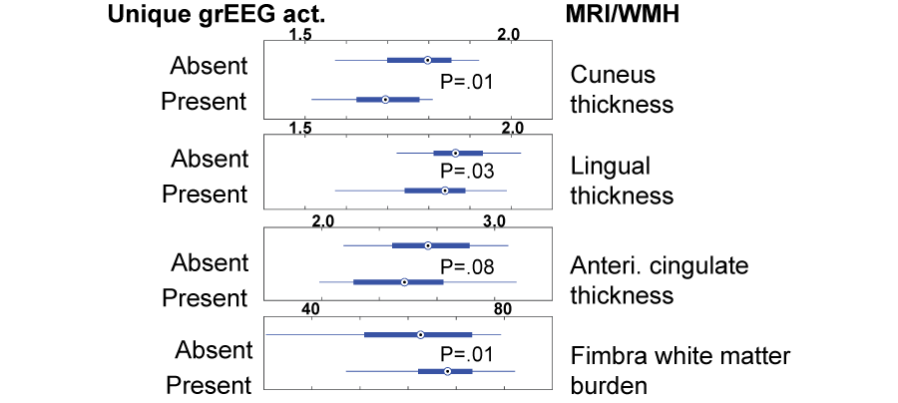
Individuals with unique grEEG pattern have smaller cuneus, lingual, ant. cingulate, and increased white matter burden of the fimbra, which connects the hippocampus. Act.: activity; ant.: anterior; Anteri.: anterior; grEEG: gait-related electroencephalographic; MRI: magnetic resonance imaging; WMH: white matter hyperintensities.

Figure 6 shows preliminary findings in support of our hypothesis that Aβ burden [90] as well as MRI-regions impacted early during AD and cerebrovascular pathology are nominally associated with the presence of unique grEEG activation. Figure 6 shows the volume and white matter differences in brain regions between participants with and without unique grEEG patterns during DTW (15/28). Further, when grouping participants in tertiles based on amyloid probability score (APS) [90]—APS accounts for Aβ 42/40 ratio, age, and *ApoE* phenotype - we find that high APS is nominally associated with the increased presence of unique grEEG activation (1st tertile: 1 out of 5; 2nd tertile: 3 out of 6; 3rd tertile: 3 out of 5).

#### MRI Image Acquisition

All images will be acquired at the Gruss Magnetic Resonance Research Center at Einstein, using a Philips 3T Elition multinuclear MRI/magnetic resonance spectroscopy system. Our noninvasive MRI imaging procedures are reliable and extensively used in aging and dementia studies. T1-weighted images will be acquired using axial 3D-magnetization-prepared rapid acquisition with gradient echo parameters over a 240 mm field of view; 1.0 mm isotropic resolution, echo time/repetition time=4.6/9.9 ms, α= 8o, and SENSE factor 2.6. 3D-FLAIR images will be acquired with repetition time/inversion time=4800/1650 ms, echo time=335 ms, 240 × 238 acquisition matrix, and 0.46 mm voxel size. Functional (resting-state and task-based), diffusion-weighted, arterial spin labeling, and susceptibility-weighted images will also be acquired as part of the parent study (for additional details see [32]). A radiologist will review each MRI scan and confirm that there are no clinically significant findings for any of the participants. WMH will be quantified from 3D-FLAIR using the lesion segmentation toolbox [91] (implemented with SPM12/MatLab). T1-weighted images will be reconstructed using FreeSurfer (version 7.2; FreeSurfer). FreeSurfer’s subcortical segmentation and cortical parcellation are comparable to manual labeling [92]. Parcellation of the T1-weighted data into anatomical brain regions is important for examining cortical thickness and volume associated with the grEEG pattern during visually perturbed walking or dual-task walking. Gray matter volumes and cortical thicknesses of 68 cortical regions will be extracted from this pipeline and entered into subsequent statistical models.

#### Statistical Plan Aim 2

##### Primary Analyses

The time-to-event analyses will be used to assess whether Aβ and WMH predict the incidence of the neural signature of CdF. Prevalent cases—that is, older adults with the unique grEEG pattern at baseline—will be excluded. Time to the neural signature will be defined as the time interval between baseline and the date when the participant demonstrates the unique grEEG pattern during the longitudinal follow-up.

##### Power Analysis

Preclinical Aβ/WMH prevalence is estimated at 24% to 45% among older adults who are cognitively unimpaired [93-95]. With a baseline sample size of 180 and a conservative attrition rate of 20%, the minimum detectable hazards ratio for incident neural signature of CdF between older adults with and without dementia-related pathology is 1.75 (assuming an event rate of 0.25) with a statistical power of 80% and a 2-sided type I error rate of 5%.

### Aim 3: Establish Associations Between Physical Inactivity and Neural Signature of CdF

#### Overview

**Figure 7:**
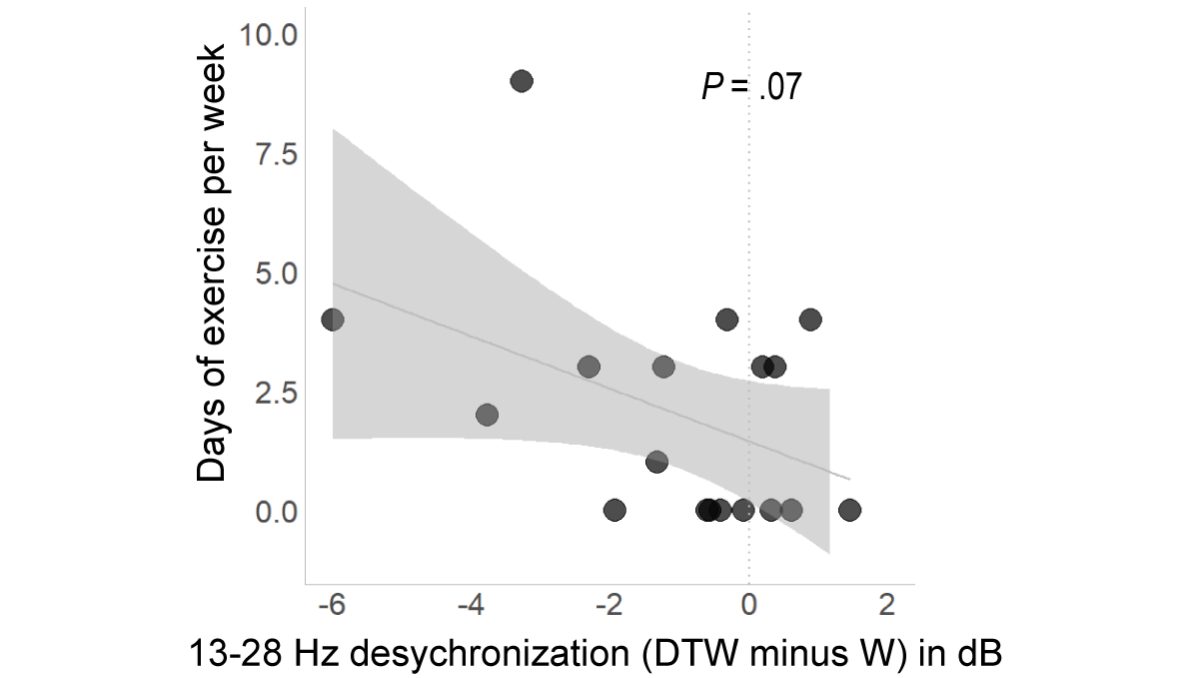
Less exercise is related to less β desynchronization during DTW. DTW: dual-task walking; W: walking.

Figure 7 shows preliminary findings in support of our prediction that less exercise (assessed with the Late Life Function and Disability Instrument [96,97]) is nominally associated with 13-28 Hz less desynchronization during DTW.

#### Statistical Plan Aim 3

##### Overview

The time-to-event analysis will be used to assess whether physical activity at baseline predicts the presence of the neural signature of CdF longitudinally.

##### Power Analysis

The minimum detectable hazards ratio for incident neural signature of CdF between older adults with less physical activity and normal level physical activity is 1.78 (assuming an event rate of 0.25) with a power of 0.8 and a 2-sided type I error rate of 5%.

## Discussion

### Principal Findings

A major effort aimed at curbing the rapid rise of dementia revolves around early intervention. To substantiate early intervention, it is crucial to enhance the accuracy of early detection. We focus on the neural signature of early limitations in CdF to improve detection. Walking is central to many instrumental and complex activities [9,10]. To determine the neural signature of CdF, our team [11-19] and others [73,87,98-104] have established the validity and reliability of neurophysiological recordings while participants are in motion. We designed novel grEEG protocols for this proposal and selected walking as an activity that, compared to other daily activities (eg, preparing a meal), allows for the use of standardized, reproducible, reliable, and well-tolerated study protocols across participants [30,39,105,106].

Our preliminary findings in 28 older adults without MCI/dementia show that reporting of CdF limitations is associated with a unique grEEG activation pattern: 13-28 Hz central gyrus desynchronization paired with 3-7 Hz fronto-medial synchronization. We term this electroencephalographic activation pattern the neural signature of CdF. This subsample also had higher Aβ burden, smaller brain volume, and WMH in regions affected early by dementia and engaged in less physical exercise.

Some limitations are important to note. The ceiling effect with a majority reporting no limitations in CdF is a concern (eg, in Marshall et al [79] study only 16% reported limitations). However, in our study, 30% of individuals reported limitations [1]. To optimize detection, we choose 2 validated and reliable instruments (self-report [107] and paper-pencil test [35] with numerous dependent variables such as self-assessment, time-to-complete, and number and type of errors to quantify performance) covering a broad range of complex daily activities. Further, different processes denoted by Aβ deposition and WMH and their respective roles in the etiology of AD and other dementias are matters of ongoing debates. The prevalence of amyloid deposition in normal aging warrants consideration of Aβ in concert with phosphorylated τ and neurofilament light assays to recognize the multitude and temporal order of pathological processes leading up to clinical signs.

In summary, establishing a noninvasive neurophysiological signature of prevalence and incidence of CdF decline will refine pre-MCI stage characterization. As our recent study shows [1], both individuals who progress and do not progress to MCI may report mild limitations in CdF during the preclinical period, though more limitations are reported in the individuals who go on to develop MCI. By establishing the clinical relevance and biological basis of the neural signature of CdF decline, we aim to improve prediction during preclinical stages of ADs and other dementias.

## Appendix

Multimedia Appendix 1 Peer review summary by NIA study section.

## Abbreviations

Aβ: amyloid-β
AD: Alzheimer disease
ADL-PI: Activities of Daily Living–Prevention Instrument
AECOM: Albert Einstein College of Medicine
APS: amyloid probability score
AT(N): amyloid-β, phosphorylated τ, neurofilament
CdF: complex daily function
ERSP: event-related spectral perturbation
grEEG: gait-related electroencephalographic
IC: independent component
IRB: institutional review board
MCI: mild cognitive impairment
MoCA: Montreal Cognitive Assessment
MRI: magnetic resonance imaging
PI: principal investigator
VSI: visual-somatosensory integration
WMH: white matter hyperintensities

## Data Availability

The data set generated in this study is available from the corresponding author upon reasonable request and with the completion of the data user agreement.
