# Correction: Linking Dementia Pathology and Alteration in Brain Activation to Complex Daily Functional Decline During the Preclinical Dementia Stages: Protocol for a Prospective Observational Cohort Study

**DOI:** 10.2196/66438

**Published:** 2024-10-21

**Authors:** Pierfilippo De Sanctis, Jeannette R Mahoney, Johanna Wagner, Helena M Blumen, Wenzhu Mowrey, Emmeline Ayers, Claudia Schneider, Natasha Orellana, Sophie Molholm, Joe Verghese

**Affiliations:** 1 Department of Neurology, Division of Cognitive and Motor Aging Albert Einstein College of Medicine Bronx, NY United States; 2 Department of Pediatrics Cognitive Neurophysiology Laboratory Albert Einstein College of Medicine Bronx, NY United States; 3 Swartz Center for Computational Neuroscience Institute for Neural Computation University of California, San Diego La Jolla, CA United States; 4 Department of Medicine (Geriatrics) Albert Einstein College of Medicine Bronx, NY United States; 5 Department of Epidemiology and Population Health Albert Einstein College of Medicine Bronx, NY United States

In “Linking Dementia Pathology and Alteration in Brain Activation to Complex Daily Functional Decline During the Preclinical Dementia Stages: Protocol for a Prospective Observational Cohort Study” (JMIR Res Protoc 2024;13:e56726), one error was noted.

In the first paragraph of the “Methods” section and in “Acknowledgments”, the grant number listed as *R01AG036921* has been revised to *R01AG075679*.

The correction will appear in the online version of the paper on the JMIR Publications website on October 21, 2024, together with the publication of this correction notice. Because this was made after submission to PubMed, PubMed Central, and other full-text repositories, the corrected article has also been resubmitted to those repositories.

