# Supplementary material for: Linking Dementia Pathology and Alteration in Brain Activation to Complex Daily Functional Decline During the Preclinical Dementia Stages: Protocol for a Prospective Observational Cohort Study
Source: JMIR Res Protoc. 2024 Jun 6;13:e56726. doi: 10.2196/56726 (PMC11190628; doi:10.2196/56726)
Supplement: Multimedia Appendix 1 [file resprot_v13i1e56726_app1.pdf]

SUMMARY STATEMENT
(Privileged Communication)

PROGRAM CONTACT:
Coryse St. Hillaire-Clarke
301-496-9350
sthillaireclacn@mail.nih.gov

Release Date: **10/26/2022**
Revised Date:

Application Number: **1 R01 AG082188-01**

Principal Investigator

DE SANCTIS, PIERFILIPPO

Applicant Organization: ALBERT EINSTEIN COLLEGE OF MEDICINE

Review Group: **ASG**
Aging Systems and Geriatrics Study Section

Meeting Date: **10/13/2022**
Council: **JAN 2023**
Requested Start: **04/01/2023**
RFA/PA: **PAR22-093**
PCC: **3DMMPCS**

Project Title: **Linking dementia pathology and alteration in brain activation to complex daily functional decline during the preclinical dementia stage**
SRG Action: **Impact Score:46 Percentile:27**
Next Steps: **Visit https://grants.nih.gov/grants/next_steps.htm**
Human Subjects: 48-At time of award, restrictions will apply
Animal Subjects: 10-No live vertebrate animals involved for competing appl.
Gender: 1A-Both genders, scientifically acceptable
Minority: 1A-Minorities and non-minorities, scientifically acceptable
Age: 8A-Only Older Adults, scientifically acceptable

Project Year	Direct Costs Requested	Estimated Total Cost
1	493,702	829,419
2	494,515	830,785
3	486,399	817,150
4	476,700	800,856
5	446,428	749,999
TOTAL	2,397,744	4,028,209

ADMINISTRATIVE BUDGET NOTE: The budget shown is the requested budget and has not been adjusted to reflect any recommendations made by reviewers. If an award is planned, the costs will be calculated by Institute grants management staff based on the recommendations outlined below in the COMMITTEE BUDGET RECOMMENDATIONS section.
NEW INVESTIGATOR

1R01AG082188-01 De Sanctis, Pierfilippo

NEW INVESTIGATOR
PROTECTION OF HUMAN SUBJECTS UNACCEPTABLE

RESUME AND SUMMARY OF DISCUSSION: The principal investigator proposed a study to test the ability of a portable electroencephalography (EEG) device to generate distinct gait-related neural signatures of early-stage mild cognitive impairment (MCI). Opinions amongst the reviewers regarding the potential impact of the application were on a broad range. One opinion was that the establishment of an MCI neural signature via this novel non-invasive EEG method would enable more substantially more efficient detection of asymptomatic cognitive decline and would have wide-scale applicability. On the other hand, the expected gains of the proposal were viewed incremental beyond the blood biomarker assessments that were included for comparison. The reviewers cited the principal investigator's rationale for investigation of the gait-related EEG signature and strong preliminary data as strengths of the approach. However, they also noted some weaknesses in the plan including potential power issues related to the low predicted (~30%) incidence of MCI in their cohort and a limited scope of AD biomarkers. All reviewers agreed that the principal investigator and their team were well-suited to carry out the proposed work, but they also expressed concern regarding absence of a team member with expertise in Alzheimer's Disease and Related Dementias. By the end of the discussion, the reviewers were unable to come to a consensus regarding the overall impact of the proposal owing to their contrasting perspectives on its significance. This difference of opinion was reflected in their final impact scores.

DESCRIPTION (provided by applicant):
Abstract: Progressive difficulty in performing everyday functional activities is a key diagnostic feature of dementia syndromes. However, not much is known about the neural signature of functional decline, particularly during the very early stages of dementia. Early intervention before overt impairment is observed offers the best hope of reducing the burdens of Alzheimer's disease and other dementias. But to justify early intervention, those at risk need to be detected earlier and more accurately. Decline in complex daily function (CdF) such as managing medications has been reported to precede impairment in basic activities of daily living (e.g., eating, dressing). Our goal is to establish the neural signature of decline in CdF during the preclinical dementia period. Gait is central to many CdF and community-based activities.2,3 Hence, to elucidate the neural signature of CdF, we validated a novel electroencephalographic (EEG) approach to measure gait-related brain activation while participants perform complex gait based functional tasks. Our hypothesis is that dementia-related pathology during the preclinical period activates a unique gait-related EEG pattern that predicts subsequent decline in CdF. We provide preliminary findings showing that older adults endorsing CdF limitations can be characterized by a unique gait-related neural signature: weaker sensorimotor and stronger motor control activation. This subsample also had smaller brain volume and WMH in regions affected early by dementia and engaged in less physical exercise. We propose a prospective observational cohort study in cognitively unimpaired older adults with and without subclinical Alzheimer's disease (plasma amyloid-β [Aβ]) and vascular (white matter hyperintensities [WMH]) pathologies. Our aims are to 1) establish the unique gait-related EEG activation as the neural signature and predictor of decline in CdF during the preclinical dementia period; 2) determine associations between dementia-related pathologies and incidence of neural signature of CdF; 3) establish associations between a dementia risk factor, physical inactivity, and the neural signature of CdF. By establishing the clinical relevance and biological basis of the neural signature of CdF decline, we aim to improve prediction during the preclinical stages of Alzheimer's diseases and other dementias. Our approach has important research and translational

implications because gait-related EEG protocols are relatively inexpensive and portable, and predicting CdF decline may have real world benefits.

PUBLIC HEALTH RELEVANCE

NARRATIVE Progressive difficulty in performing everyday functional activities is a key diagnostic feature of dementia syndromes, but little is known about the neural signature of functional decline, particularly during the very early stages of dementia. We apply a novel neurophysiological approach to measure brain activation during complex gait based functional tasks to establish a biomarker and predictor of functional decline during the preclinical dementia period before overt impairment is observed. Establishing the clinical relevance and biological basis of the neural signature of functional decline will improve early detection and maintain functional abilities, independence, and quality of life in older adults at-risk for dementia.

CRITIQUE 1

Significance: 5
Investigator(s): 3
Innovation: 3
Approach: 5
Environment: 1

Overall Impact: This application investigates the interesting hypothesis that preclinical ADRD pathology results in a gait-related EEG pattern that predicts development of impairments in complex daily functions (CdF). The premise is built upon a reasonably strong literature indicating that CdF changes are an early event that occur prior to MCI. As such, identifying the unique grEEG pattern may not only identify persons at-risk for CdF but also prove useful in further characterizing transitions from normal cognition to MCI and dementia as well as assessing modulators of disease progression. This potential significance is counterbalanced by concerns regarding the clinical utility of the measure, its relative value in comparison to other biomarkers, and the likelihood for translation given the approaches currently used. The investigative team is excellent overall. The PI is supported by several Co-Is with overlapping and complementary areas of expertise in most areas relevant to the proposed work. Importantly, there exists a robust history of funding and collaboration within the team that is supportive for successful completion. The team would be improved by inclusion of additional expertise in AD, especially with regards to Aim 2 studies. The design of the study has strengths in terms of its conceptual focus on early stages of preclinical AD and the leveraging of an existing, funded study to augment the number of subjects. There are several concerns with the approach, particularly with Aim 2 studies that will examine the relationship between AD-related pathology markers and subsequent development of CdF. In particular, there is a low number of indicators with other MR and plasma biomarkers readily available. Also of concern is the fairly low proportion of cognitively normal persons that will present with both CdF issues and AD-related pathology. These and other noted weaknesses in the approach as well as the stated concerns in significance and impact were important considerations in the overall scoring.

1. Significance:

Strengths

- As declines in performance of complex daily activities occurs in preclinical phases of AD, its early detection has potential benefits in terms of both identifying at-risk persons and defining a measure that can be used to assess modulation of disease progression.

- The findings may better characterize subpopulations and their trajectories of decline in the transitions from normal cognition – MCI – dementia.

Weaknesses

- The utility of this measure for clinically meaningful prevention or improvement in outcomes is unclear.
- If minimally invasive AD biomarkers such as plasma Abeta are strongly associated with and predict subsequent CdF (as hypothesized), then what is the need for an EEG-based measure?
- Some concern about the translational potential given the complexity of the EEG set-up and that the grEEG signature is based upon gait tasks that appear to require a special lab environment.

2. Investigator(s):

Strengths

- Very strong investigative team with respect to most key areas.
- PI is an early-stage investigator with strengths in studying motor function with neural activation, areas highly relevant to the proposed work.
- Co-I Verghese provides essential expertise in mobility and its clinical assessment and serves as the K award mentor for the PI.
- PI Mahoney has a newly funded project for which subjects will be cross enrolled into the proposed study.
- Other Co-Is bring additional expertise in EEG, multi-sensory processing, neuroimaging, gait control and biostatistics.
- Excellent history of collaboration among the investigative team.

Weaknesses

- Clear need for more expertise in Alzheimer's disease.

3. Innovation:

Strengths

- Some conceptual novelty in using a neurophysiological measure as an early indicator of functional and cognitive decline.
- Focus on preclinical stages of dementia.
- A portable EEG approach has some translational potential though not in its current state.

Weaknesses

- None noted.

4. Approach:

Strengths

- Preliminary findings are generally consistent with hypothesized grEEG differences in persons with mild differences in cognition as well as those with trends of early impairments in CdF.

- Leverages another study for 70 of 180 participants that shares common protocols and outcome measures.
- Emphasis on early stages of declines.

Weaknesses

- Besides Abeta, standard A/T/N plasma biomarkers are not included.
- Related concern that only two pathology markers (Abeta, WMH) are assessed as predictors of CdF.
- As low number of participants with CdF is possible/likely and power analyses indicate a need for >50 participants, there are concerns about subject retention especially over 3–4-year period.
- The feasibility of the Aim 2 studies is a concern given the sample size. The power and statistical analyses in Aim 2 do not address the proportion of the cognitively normal participants that also exhibit significant abnormalities in WMH and plasma Abeta at baseline such that there will be sufficient N that also develop CdF impairments (which is a minority of subjects).
- For Aim 3, physical activity several years if not decades before study enrollment may reasonably affect pathology and the investigated relationships but is not considered.
- As power appears to be limited, the ability to consider sex differences is limited.

5. Environment:

Strengths

- The facilities and scientific environment at Albert Einstein are outstanding.
- Excellent project-relevant resources and equipment including capabilities including the Cognitive Neurophysiology Lab (EEG), DCMA (gait), and neuroimaging center.

Weaknesses

- Unusual that protocols for plasma analysis are listed under Equipment.

Protections for Human Subjects:

Acceptable Risks and/or Adequate Protections

Data and Safety Monitoring Plan (Applicable for Clinical Trials Only):

Not Applicable (No Clinical Trials)

Inclusion Plans:

- Sex/Gender: Distribution justified scientifically
- Race/Ethnicity: Distribution justified scientifically
- For NIH-Defined Phase III trials, Plans for valid design and analysis: Not applicable
- Inclusion/Exclusion Based on Age: Distribution justified scientifically

Vertebrate Animals:

Not Applicable (No Vertebrate Animals)

Biohazards:
Not Applicable (No Biohazards)

Applications from Foreign Organizations:
Not Applicable (No Foreign Organizations)

Select Agents:
Not Applicable (No Select Agents)

Resource Sharing Plans:
Acceptable

Authentication of Key Biological and/or Chemical Resources:
Not Applicable (No Relevant Resources)

Budget and Period of Support:
Recommend as Requested

CRITIQUE 2

Significance: 2
Investigator(s): 1
Innovation: 1
Approach: 4
Environment: 1

Overall Impact: The application proposes to investigate brain functions during active walking with or without a cognitive task as marker for cognitive decline and daily functions. The proposed studies will also characterize older adults' neuropsychological profiles, identify risk factors including social determinants of health. Gaiting has become popular indicator for early detection of dementia risk. PI is an impressive early career investigator who a K-award that is related to the current proposal. The proposed study combining gait and EEG is highly significant and innovative. PI and the team have developed the feasibility of a novel EEG mobile brain-body imaging. The proposed scientific premise was based on strong preliminary results establishing body-brain relations using EEG based monitoring system that employs 3-D infrared camera for capturing gait, posture, strike, and falls during ambulation is high impact. The Gait-related EEG also was mapped with brain functions using MRI technologies, which has a body of literature. Minor concern is lack of consideration of various subtypes of ADRD that differ in cognitive motor functions. Overall, EEG-based brain signatures are powerful translational method to stimulate new directions in the field. The portable device has great potential for large-scale screening for dementia risk in normal preclinical older adults. Combining EEG during walking, the proposal is expected to lead to high-impact predictors in risk of daily functioning deficits in older adults long before diagnosis.

1. Significance:

Strengths

- Establishing body-brain relations using EEG based monitoring system that employs 3-D infrared camera for capturing gait, posture, strike, and falls during ambulation is highly significant.
- The proposed study is expected to lead to predicting potential risk in deficits of daily functioning in older adults before diagnosis.

Weaknesses

- None noted.

2. Investigator(s):

Strengths

- PI is an impressive early career investigator. PI has a K award that is related to the current proposal.
- PI, Co-I (K award mentor) and other outstanding team of researchers brought complimentary skills and experience.

Weaknesses

- There is no notable weakness.

3. Innovation:

Strengths

- EEG can be measured during ambulation. EEG-based brain signatures are powerful new method to and will enable new directions in the field. Neuroimaging methodologies such as Magnetic Resonance Imaging requires holding still.
- Exploration of the results of the novel system is expected to yield numerous advances.

Weaknesses

- None noted.

4. Approach:

Strengths

- Strong pilot results that weaker central gyrus beta (13-28 Hz) de-synchronization paired with stronger frontal theta (3-7 Hz) synchronization in MOCA score 22-26, but not in those 27-30.
- Dual walking task EEG and simple walking related EEG are good design.
- Linking gait-related EEG indicators with more expensive methods MRI (e.g., white matter hyperintensity).
- The experimental design is comprehensive.
- Well planned key measures and procedures during each visit.
- Simultaneous recording methods are strength.

Weaknesses

- Hypotheses of daily functions were broadly defined as same as MoCA cognitive screening test. There is limited rigor of examine relations of EEG markers and specific cognitive functions, e.g., working memory, executive function, or language.
- Cognitively normal individuals using a cut score of ≥ 22 on the MOCA does not distinguish mild cognitive impairments induced by various subtypes of ADRD (e.g., Lewy body dementia).

5. Environment:

Strengths

- The existing set up and support of the Albert Einstein University allows sustain of the research.

Weaknesses

- None noted.

Protections for Human Subjects:

Acceptable Risks and/or Adequate Protections

- Low risk and established protocol.

Data and Safety Monitoring Plan (Applicable for Clinical Trials Only):

Not Applicable (No Clinical Trials)

Inclusion Plans:

- Sex/Gender: Distribution justified scientifically
- Race/Ethnicity: Distribution justified scientifically
- For NIH-Defined Phase III trials, Plans for valid design and analysis: Not applicable
- Inclusion/Exclusion Based on Age: Distribution justified scientifically
- Sex as biological variable, age, race/ethnicity, and SES variables were included

Vertebrate Animals:

Not Applicable (No Vertebrate Animals)

Biohazards:

Acceptable

- Protocols are in place.

Applications from Foreign Organizations:

Not Applicable (No Foreign Organizations)

Select Agents:

Not Applicable (No Select Agents)

Resource Sharing Plans:

Acceptable

- In place.

Authentication of Key Biological and/or Chemical Resources:

Acceptable

Budget and Period of Support:

Recommend as Requested

CRITIQUE 3

Significance: 6
Investigator(s): 3
Innovation: 4
Approach: 6
Environment: 2

Overall Impact: This R01 submissions is from a new investigator, Dr. De Sanctis, at NYU. This proposal will refine an EEG-based neural signature of complex daily functioning by enrolling 180 cognitively unimpaired older adults who will complete EEG while completing walking tasks. The prevalence of this EEG neural signature of CdF will be determined (aim 1), and the signature will be related to plasma abeta and WMH on brain MRI (aim 2). Lastly, the signature will be related to physical activity (subjective and actigraphy). While developing novel ways to detect preclinical disease is important, one overall concern with this proposal is significance and whether the proposed study will advance knowledge in the field. Creating an EEG-based signature may not be generalizable, and it is not clear if it is any better than measuring self-reported difficulty on complex tasks. Other weaknesses noted are related to approach, including how the signature is defined, vague descriptions on how the signature will be compared to other brain health metrics. Lastly, the reliance on plasma abeta is a weakness given the novelty and lack of established validity of this metric to determine biomarker status.

1. Significance:

Strengths

- None Noted.

Weaknesses

- None Noted.

2. Investigator(s):

Strengths

- None Noted.

Weaknesses

- None Noted.

3. Innovation:

Strengths

- None Noted.

Weaknesses

- None Noted.

4. Approach:

Strengths

- None Noted.

Weaknesses

- None Noted.

5. Environment:

Strengths

- None Noted.

Weaknesses

- None Noted.

Protections for Human Subjects:

Unacceptable Risks and/or Inadequate Protections

There seems to be a high risk of falls with the proposed study methods, with no preliminary data supporting the safety with older adults.

Data and Safety Monitoring Plan (Applicable for Clinical Trials Only):

Not Applicable (No Clinical Trials)

Inclusion Plans:

- Sex/Gender: Distribution justified scientifically
- Race/Ethnicity: Distribution justified scientifically
- For NIH-Defined Phase III trials, Plans for valid design and analysis: Not applicable
- Inclusion/Exclusion Based on Age: Distribution justified scientifically

Vertebrate Animals:

Not Applicable (No Vertebrate Animals)

Biohazards:

Not Applicable (No Biohazards)

Applications from Foreign Organizations:
Not Applicable (No Foreign Organizations)

Select Agents:
Not Applicable (No Select Agents)

Resource Sharing Plans:
Acceptable

Authentication of Key Biological and/or Chemical Resources:
Unacceptable

- Is authentication needed for the EEG metrics? If not, this section would not be applicable.

Budget and Period of Support:
Recommend as Requested

THE FOLLOWING SECTIONS WERE PREPARED BY THE SCIENTIFIC REVIEW OFFICER TO SUMMARIZE THE OUTCOME OF DISCUSSIONS OF THE REVIEW COMMITTEE, OR REVIEWERS' WRITTEN CRITIQUES, ON THE FOLLOWING ISSUES:

PROTECTION OF HUMAN SUBJECTS: UNACCEPTABLE, There seems to be a high risk of falls with the proposed study methods, with no preliminary data supporting the safety with older adults.

INCLUSION OF WOMEN PLAN: ACCEPTABLE

INCLUSION OF MINORITIES PLAN: ACCEPTABLE

INCLUSION ACROSS THE LIFESPAN: ACCEPTABLE

COMMITTEE BUDGET RECOMMENDATIONS: The budget was recommended as requested.

Footnotes for 1 R01 AG082188-01; PI Name: De Sanctis, Pierfilippo

NIH has modified its policy regarding the receipt of resubmissions (amended applications).See Guide Notice NOT-OD-18-197 at https://grants.nih.gov/grants/guide/notice-files/NOT-OD-18-197.html. The impact/priority score is calculated after discussion of an application by averaging the overall scores (1-9) given by all voting reviewers on the committee and multiplying by 10. The criterion scores are submitted prior to the meeting by the individual reviewers assigned to an application, and are not discussed specifically at the review meeting

or calculated into the overall impact score. Some applications also receive a percentile ranking. For details on the review process, see http://grants.nih.gov/grants/peer_review_process.htm#scoring.

MEETING ROSTER

Aging Systems and Geriatrics Study Section
Brain Disorders and Clinical Neuroscience Integrated Review Group
CENTER FOR SCIENTIFIC REVIEW
ASG
10/13/2022 - 10/14/2022

Notice of NIH Policy to All Applicants: Meeting rosters are provided for information purposes only. Applicant investigators and institutional officials must not communicate directly with study section members about an application before or after the review. Failure to observe this policy will create a serious breach of integrity in the peer review process, and may lead to actions outlined in NOT-OD-22-044 at https://grants.nih.gov/grants/guide/notice-files/NOT-OD-22-044.html, including removal of the application from immediate review.

CHAIRPERSON(S)

BAXTER, MARK G, PHD
PROFESSOR
DEPARTMENT OF PATHOLOGY
SECTION OF COMPARATIVE MEDICINE
SCHOOL OF MEDICINE
WAKE FOREST UNIVERSITY
WINSTON-SALEM, NC 27157

MEMBERS

AZCARATE-PERIL, M. ANDREA, PHD *
ASSOCIATE PROFESSOR
DEPARTMENT OF MEDICINE
UNIVERSITY OF NORTH CAROLINA
CHAPEL HILL, NC 27599

COLMAN, RICKI J, PHD
ASSOCIATE PROFESSOR
DEPARTMENT OF CELL AND REGENERATIVE BIOLOGY
NATIONAL PRIMATE RESEARCH CENTER
UNIVERSITY OF WISCONSIN, MADISON
MADISON, WI 53715

FARRELL, KURT WILLIAM, PHD *
ASSISTANT PROFESSOR
DEPARTMENT OF PATHOLOGY
ICAHN SCHOOL OF MEDICINE AT MOUNT SINAI
NEW YORK, NY 10029

GAO, SUJUAN, PHD
PROFESSOR
DEPARTMENT OF BIOSTATISTICS
SCHOOL OF MEDICINE AT INDIANA UNIVERSITY
INDIANAPOLIS, IN 46202

GIFFORD, KATHERINE A, PSYD *
ASSISTANT PROFESSOR
DEPARTMENT OF NEUROLOGY/COGNITIVE DISORDERS
SCHOOL OF MEDICINE AT VANDERBILT UNIVERSITY
NASHVILLE, TN 37232-0011

GRIMALDI, DANIELA, MD, PHD *
DEPARTMENT OF NEUROLOGY
FEINBERG SCHOOL OF MEDICINE
NORTHWESTERN UNIVERSITY
EVANSTON, IL 60208

HEFFNER, KATHI L, PHD *
PROFESSOR
DEPARTMENTS OF NURSING, MEDICINE, AND PSYCHIATRY
UNIVERSITY OF ROCHESTER MEDICAL CENTER
SCHOOL OF MEDICINE AND DENTISTRY
ROCHESTER, NY 14642

JIANG, YANG, PHD *
PROFESSOR
DEPARTMENT OF BEHAVIORAL SCIENCE
COLLEGE OF MEDICINE
UNIVERSITY OF KENTUCKY
LEXINGTON, KY 40536

KATZEL, LESLIE I, MD, PHD
ASSOCIATE PROFESSOR
DIVISION OF GERONTOLOGY
SCHOOL OF MEDICINE
UNIVERSITY OF MARYLAND BALTIMORE
BALTIMORE, MD 21201

LEVER, TERESA E, PHD *
ASSOCIATE PROFESSOR
OTOLARYNGOLOGY-HEAD AND NECK SURGERY
SCHOOL OF MEDICINE
UNIVERSITY OF MISSOURI
COLUMBIA, MO 65212

LU, HANZHANG, PHD *
PROFESSOR
DEPARTMENT OF RADIOLOGY
SCHOOL OF MEDICINE
JOHNS HOPKINS UNIVERSITY
BALTIMORE, MD 21287

MASTERNAK, MICHAL MATEUSZ, PHD
ASSOCIATE PROFESSOR
DEPARTMENT OF METABOLIC AND CARDIOVASCULAR SCIENCE
BURNETT SCHOOL OF BIOMEDICAL SCIENCES
UNIVERSITY OF CENTRAL FLORIDA
ORLANDO, FL 32827

MCCULLY, KEVIN K, PHD *
PROFESSOR
DEPARTMENT OF KINESIOLOGY
UNIVERSITY OF GEORGIA
ATHENS, GA 30602

MOLINA, ANTHONY J, PHD
ASSOCIATE PROFESSOR
DEPARTMENT OF MEDICINE
UNIVERSITY OF CALIFORNIA, SAN DIEGO
SAN DIEGO, CA 92093

PEREZ, ADRIANA, PHD
PROFESSOR
DEPARTMENT OF BIOSTATISTICS AND DATA SCIENCE
SCHOOL OF PUBLIC HEALTH
UNIVERSITY OF TEXAS HEALTH SCIENCE CENTER, HOUSTON
AUSTIN, TX 78701

PICCIO, LAURA, MD *
ASSISTANT PROFESSOR
DEPARTMENT OF NEUROLOGY
SCHOOL OF MEDICINE
WASHINGTON UNIVERSITY
SAINT LOUIS, MO 63110

PIKE, CHRISTIAN J, PHD
PROFESSOR
LEONARD DAVIS SCHOOL OF GERONTOLOGY
UNIVERSITY OF SOUTHERN CALIFORNIA
LOS ANGELES, CA 90089

PIVA, SARA R, PHD *
PROFESSOR
DEPARTMENT OF PHYSICAL THERAPY
UNIVERSITY OF PITTSBURGH
PITTSBURGH, PA 15260

PRICE, CATHERINE E, PHD
ASSOCIATE PROFESSOR
DEPARTMENT OF CLINICAL AND HEALTH PSYCHOLOGY
UNIVERSITY OF FLORIDA
GAINESVILLE, FL 32610

SAUNDERS, MILDA RENNE, AB, MPH, MD *
ASSISTANT PROFESSOR
SECTION OF GENERAL INTERNAL MEDICINE
THE UNIVERSITY OF CHICAGO
CHICAGO, IL 60637

TARANTINI, STEFANO, PHD *
ASSISTANT PROFESSOR
STEPHENSON CANCER CENTER
CENTER FOR GEROSCIENCE AND HEALTHY BRAIN AGING
UNIVERSITY OF OKLAHOMA HEALTH SCIENCES CENTER
OKLAHOMA CITY, OK 73104

TARAWNEH, RAWAN, MD *
ASSOCIATE PROFESSOR
DEPARTMENT OF NEUROLOGY
UNIVERSITY OF NEW MEXICO
ALBUQUERQUE, NM 87106

TERRANDO, NICCOLO, PHD
ASSOCIATE PROFESSOR
DEPARTMENT OF ANESTHESIOLOGY
DUKE UNIVERSITY
DURHAM, NC 27710

TOTH, MICHAEL J, PHD *
PROFESSOR
DEPARTMENT OF MEDICINE
UNIVERSITY OF VERMONT
BURLINGTON, VT 05405

WILLIS, ALLISON, MD
ASSOCIATE PROFESSOR
DEPARTMENT OF NEUROLOGY
UNIVERSITY OF PENNSYLVANIA
PHILADELPHIA, PA 19104

ZHANG, RONG, PHD
PROFESSOR
DEPARTMENT OF NEUROLOGY AND NEUROTHERAPEUTICS
UNIVERSITY OF TEXAS SOUTHWESTERN MEDICAL CENTER
DALLAS, TX 25231

MAIL REVIEWER(S)

BARRETT, FREDERICK STREETER, PHD
ASSOCIATE PROFESSOR
DEPARTMENT OF PSYCHIATRY AND BEHAVIORAL SCIENCES
SCHOOL OF MEDICINE
JOHNS HOPKINS UNIVERSITY
BALTIMORE, MD 21224

BUFORD, THOMAS W, PHD
PROFESSOR
DEPARTMENT OF MEDICINE
THE UNIVERSITY OF ALABAMA AT BIRMINGHAM
BIRMINGHAM, AL 35294

ZUWALA, ROBIN M, PHD
ASSOCIATE PROFESSOR
DEPARTMENT OF INTERNAL MEDICINE
RUSH UNIVERSITY
CHICAGO, IL 60612

SCIENTIFIC REVIEW OFFICER

BANNISTER, ROGER ALAN, PHD
SCIENTIFIC REVIEW OFFICER
CENTER FOR SCIENTIFIC REVIEW
NATIONAL INSTITUTES OF HEALTH
BETHESDA, MD 20892

EXTRAMURAL SUPPORT ASSISTANT

KELLY, COURTNEY LYNN
EXTRAMURAL SUPPORT ASSISTANT
CENTER FOR SCIENTIFIC REVIEW
NATIONAL INSTITUTES OF HEALTH
BETHESDA, MD 20892

OTHER REVIEW STAFF

ZHAO, WEI-QIN, PHD
CHIEF
AGING AND NEURODEGENERATION REVIEW BRANCH (AN)
CENTER FOR SCIENTIFIC REVIEW
NATIONAL INSTITUTES OF HEALTH
BETHESDA, MD 20892

* Temporary Member. For grant applications, temporary members may participate in the entire meeting or may review only selected applications as needed.

Consultants are required to absent themselves from the room during the review of any application if their presence would constitute or appear to constitute a conflict of interest.
